# The role of the DC component in human perception of AC–DC hybrid electric fields and a comparison with the AC component

**DOI:** 10.1038/s41598-023-43556-2

**Published:** 2023-09-28

**Authors:** Michael Kursawe, Andrea Kaifie, Julia Krabbe, Simon Kimpeler, Ralph Kühn, Thomas Kraus, Kathrin Jankowiak

**Affiliations:** 1 Research Center for Bioelectromagnetic Interaction (femu), Institute for Occupational, Social and Environmental Medicine, Uniklinik RWTH Aachen University, Pauwelsstraße 30, 52074 Aachen, Germany; 2 Institute for Occupational, Social and Environmental Medicine, Uniklinik RWTH Aachen University, Pauwelsstraße 30, 52074 Aachen, Germany; 3 Institute for High Voltage Equipment and Grids, Digitalization and Power Economics, RWTH Aachen University, Aachen, Germany

**Keywords:** Human behaviour, Energy infrastructure

## Abstract

As part of the energy transition in Germany, high-voltage overhead power lines will be operated using hybrid systems that combine alternating and direct current (AC and DC). The degree to which humans perceive hybrid electric fields (EFs) is dependent on the proportion of both EF types. To investigate the impact of the DC component, a study assessed 49 participants with above-average EF detection ability under conditions with a low DC component of 1–4 kilovolts per meter (kV/m) and varying AC EFs between 1 and 14 kV/m. The detection thresholds of combined AC/DC EFs decreased with an increase in the DC component and ranged from 9.6 to 6.83 kV/m on average for the group. The results suggest that even minor variations in the DC component significantly affect human perception of hybrid EFs. These findings complement the results of an earlier study that investigated the AC component in hybrid EFs. Correlational analyses of both studies demonstrated the reliability of participants' performance. This study contributes to our understanding of EF-related effects on human perception and can aid in the planning of energy transmission near areas where humans work or live.

## Introduction

It is well documented that humans are able to consciously perceive electric fields (EFs) emitted by high-voltage overhead power lines under real^1^ and laboratory conditions^2,3^. Human EF perception processes in the context of 50/60 Hz alternating current (AC) transmission are well described^4^, as this is the standard transmission technique used worldwide. In Germany, the goal of decarbonization is an integral part of the energy transition. However, a large distance between electric energy production site and consumption areas can make AC-high-voltage overhead transmission cost-inefficient^5^. For example, when electric energy is produced by windfarms in the North Sea and needed in western or southern Germany, alternative transmission techniques become necessary. Thus, direct current (DC) transmission will be used, which is low-loss^5^. How DC EFs are perceived by humans was investigated in the past^2,3,6^. Furthermore, some cases require mounting both AC and DC overhead power lines on the same pylon, leading to hybrid EFs in the immediate vicinity. Compared to single EFs, hybrid EFs produce a more intense human perception, which is highly influenced by the proportion of both field types^1–3,7^. Even minor changes in this proportion can significantly alter perception^7^ and should be taken into account during the design process of high-voltage overhead power lines.

Although transmission systems with combined AC–DC overhead power lines are under construction in Germany, no reference value exists related to the emitted EF. Reference levels of 5 kV/m for AC EF (referring to the frequency range of 25–300 Hz) and 20 kV/m in the frequency range of 1–8 Hz are suggested by the ICNIRP^8^. However, no recommendation for DC EFs is provided by the ICNIRP. According to the Institute of Electrical and Electronic Engineers, a maximum of 10 kV/m should not be exceeded in static EFs^9^.

Human perception of EFs can be quantified by individual detection performance of a set of predefined EF strengths using signal detection theory^10^. The detection threshold refers to the EF strength, where a person can successfully detect the EF from a set of signal and non-signal situations. For AC EF, detection thresholds in whole-body investigations were found to be 14.16 kV/m (RMS value), whereas DC EF detection thresholds were somewhat higher with 18.69 kV/m^3^. Blondin et al.^6^ showed DC EF detection thresholds between 40.1 and 45.1 kV/m, while they observed a subgroup of one third having no perception in DC EFs of up to 50 kV/m at all.

When exposed to a combination of AC and DC EF, people rated an EF combination of 15 kV/m DC and 5 kV/m AC as just perceptible^1^. In a laboratory study, participants showed a detection threshold of 6.76 kV/m DC, while a constant AC EF of 4 kV/m was present^3^. Based on these results, Jankowiak et al.^7^ investigated in detail how the AC component modulated the detection performance of hybrid EF in 51 highly sensitive participants. Four conditions were generated with a constant AC EF of 1, 2, 3 or 4 kV/m and varying DC EFs between 1 and 16 kV/m. The authors computed total EF detection thresholds per condition by combining AC and DC EF strengths^7^. Detection thresholds of total EF strengths decreased with 8.89 kV/m, 7.82 kV/m, 6.48 kV/m, and 5.7 kV/m in conditions, where 1, 2, 3, and 4 kV/m AC was present, respectively. Therefore, a larger proportion of AC EF leads to decreased detection thresholds in hybrid EF^7^.

Although human perception of EFs is well documented, underlying biological mechanisms are not completely understood. Most research focused on hair characteristics or even its absence. Reilly^4^ explained AC EF perception based on electric charge of the skin leading to a push-away-mechanism of single hairs. Resulting vibrations would thus produce a perception at the skin surface. Further it was shown that detection thresholds increased when hairs at the fore arm were removed^11,12^. The electric force on hairs of superimposed AC/DC EFs was shown to vary with ratio of both field types^13^. In general, hair and skin hydration level influenced EF perception^12,14^, whereas others did not find a correlation with skin hydration level^3,7^.

The current study aimed to add information to the question, how small variations in one EF component influence human perception of hybrid EFs by focusing on DC. Expanding on the results of Jankowiak et al.^7^ showing a decrease of total EF detection thresholds with an increasing AC component, the current study examined the impact of the DC component on human EF perception. Therefore, 49 participants with high detection ability were investigated using a design that combines small variations of the DC component with different AC EFs.

## Methods

### Participants

Forty-nine participants (30 female, 19 male) were included in this study, aged between 24 and 79 years (mean age: 51 years). All participants already took part in an earlier investigation^3^, where they reached a specific criterion of successful detection of a hybrid EF with 4 kV/m AC and 2 kV/m DC. This selection process led to a homogenous group with respect to the detection performance. Based on previous research^7^, we had a test power of > 0.99 to find a large effect with respect to the analysis of detection thresholds.

Exclusion criteria were electronic implants or not removable piercings, self-reported electromagnetic hypersensitivity, neurological or psychiatric disorders, claustrophobia, or severe skin diseases. In addition to a detailed medical anamnesis conducted by a physician, participants with signs of infection, skin abnormalities, and neurological or psychiatric disorders were excluded. To exclude a pregnancy, all female participants in childbearing age conducted a urinary β-hCG test. Participants were screened for exclusion criteria, examined and informed before inclusion and signed an informed consent. An expense allowance of 100 Euros was paid to participants afterwards. The study was approved by the Ethics Committee of the Medical Faculty of the RWTH Aachen (EK435/21) and complied with the Declaration of Helsinki.

### Exposure laboratory and psychophysiological testing

EF perception investigations were conducted in a specialized exposure laboratory at the University Hospital RWTH Aachen. Facility, technical setup, security systems, and calibration methods of the high-voltage circuit are described in detail by Jankowiak et al.^2^. The study was designed as a double-blind investigation with the participant sitting in the exposure laboratory and the investigator sitting in an adjacent room. To mask possible noise from any part of the field generating system, a 61.7 dB(A) white noise was played during the experimental sessions. While participants were placed on a centralized wooden chair, electrodes integrated in the ceiling of the exposure laboratory were energized via a 50 kV AC transformer (50 Hz) and a 200 kV DC power source. The distribution of EFs at the center of exposure laboratory was kept homogenous through 14 grading electrodes mounted at the wall interconnected via ohmic-capacitive grading units. The floor of the exposure laboratory is connected to ground. All EF combinations used in the study were calibrated prior to the start. A trained technician observed proper technical functioning at any time during perception testing.

To ensure participants safety, ankle electrodes were connected to the ground at any time. Additionally, two cameras filmed participants during testing and a constant connection to the operator was realized via an intercom system. A light barrier system generated a space of about 2 × 2 × 1.67 m, wherein the chair was located. Passing one of the light barriers stopped testing immediately and led to a shut-down including grounding of the high-voltage system within 180 ms. A redundant safety system includes a belt with contact plugs as well as an emergency stop.

EF and sham exposures were intermixed randomly, and participants had to indicate whether they perceived an EF or not. To determine individual sensitivity towards a given EF strength, positive responses to EF exposure (hit) and positive responses to sham exposures (false alarm) were taken into account. According to Green and Swets^10^, the individual sensitivity was calculated by *d*′ = z(hit) − z(false alarm). A *d*′ ≥ 1 indicates a distance of at least one standard deviation between the distributions of hit and false alarm responses referring to a successful EF detection. Values of 2 ≤ *d*′ < 3 indicate good sensitivity and values of *d*′ ≥ 3 are associated with excellent EF detection performance^10^.

### Test protocol

Laboratory environmental condition was set to 22 °C and 50% relative humidity. After participants were examined and informed by a physician at 8:30 A.M., they received an introduction in the laboratory including all safety systems. Four test conditions were determined with a constant DC EF of 1, 2, 3, or 4 kV/m and varying AC EF strengths between 1 and 14 kV/m (see Table 1). Each test condition was divided into two sessions. In total, eight sessions were conducted, each lasting 15 min. One session consisted of 40 trials, 50% of which were sham trials, randomly intermixed with exposure trials. A trial started with a 3 seconds (s) lasting onset period, where EF was increased to the predefined strength. Thereafter, a 5 s period was inserted to “perceive” the EF followed by a 4 s lasting response period. At the wall in front of the participants, the question appeared “do you perceive an electric field?” together with four response alternatives “yes – certain”, “yes – uncertain”, “no – uncertain”, “no – certain”. After participants’ response, the EF decreased and grounding of the high voltage system was done, which lasted 9 s. The same timing was applied in sham trials without any EF exposure. After the first session, a 1–2 min break was necessary for preparing the next session. After completing two sessions, a break of 15 min was inserted where participants could relax. At 12:30 P.M. the study was finished, and participants were asked if they experienced any unusual health issues. In this case, a physician was available for a checkup. None of the participants experienced any unusual health symptoms.

**Table 1:** Four test conditions applied in the study. Total EF strengths were calculated by ${E}_{\text{RMS}}=\sqrt{{{E}_{\text{DC}}}^{2}+{{E}_{\text{AC}}}^{2}}$*.* Each total EF strength was presented eight times leading to 40 exposure trials and 40 sham trials per test condition.

	DC EF strength (kV/m)	AC EF strength (kV/m)	Total EF strength (kV/m)
Condition 1	1	1, 2, 4, 8, 14	1.41, 2.24, 4.12, 8.06, 14.04
Condition 2	2	1, 2, 4, 8, 14	2.24, 2.83, 4.47, 8.25, 14.14
Condition 3	3	1, 2, 4, 8, 14	3.16, 3.61, 5.00, 8.54, 14.32
Condition 4	4	1, 2, 4, 8, 14	4.12, 4.47, 5.66, 8.94, 14.56

### Data processing and analysis

To quantitatively evaluate participants ability to detect EFs, the sensitivity index *d*′ was calculated by *d*′ = z(hit) − z(false alarm). Since z-values cannot be calculated when relative detection performance was 0 or 1, a log-linear transformation of hits and false alarm rates was done^15^. Thereafter, *d*′-values were entered into a 4 × 5 repeated measures analysis of variance (rm ANOVA) with factors *DC EF strength* (1, 2, 3, 4 kV/m) and *AC EF strength* (1, 2, 4, 8, 14 kV/m). An α-level of *p* = 0.05 was accepted for significance level and partial eta squared (η_p_^2^) are provided to estimate effect sizes. Additionally, individual detection thresholds were calculated based on total EF strengths with ${E}_{\text{RMS}}=\sqrt{{{E}_{\text{DC}}}^{2}+{{E}_{\text{AC}}}^{2}}$, where ${E}_{\text{AC}}$ refers to the RMS value. Thereafter, individual psychometric functions for every test condition were used to determine the individual detection threshold referring to *d*′ = 1. This was only possible when trajectory was plausible i.e., an increase of *d*′ with increasing EF strengths. For example, when in test condition 1 a *d*′ ≥ 1 was found in total EF strengths of 1.41 and 2.24 kV/m but a *d*′ < 1 was observed when 4.12 kV/m was applied, the trajectory was classified as implausible. Furthermore, when participants were not able to detect any EF e.g., in test condition 1, no detection threshold could be computed. Individual detection thresholds from participants taking part in the current study as well as in our former study^7^ were correlational analyzed. Pearson coefficient *r* was calculated for detection thresholds in every test condition.

## Results

**Figure 1:**
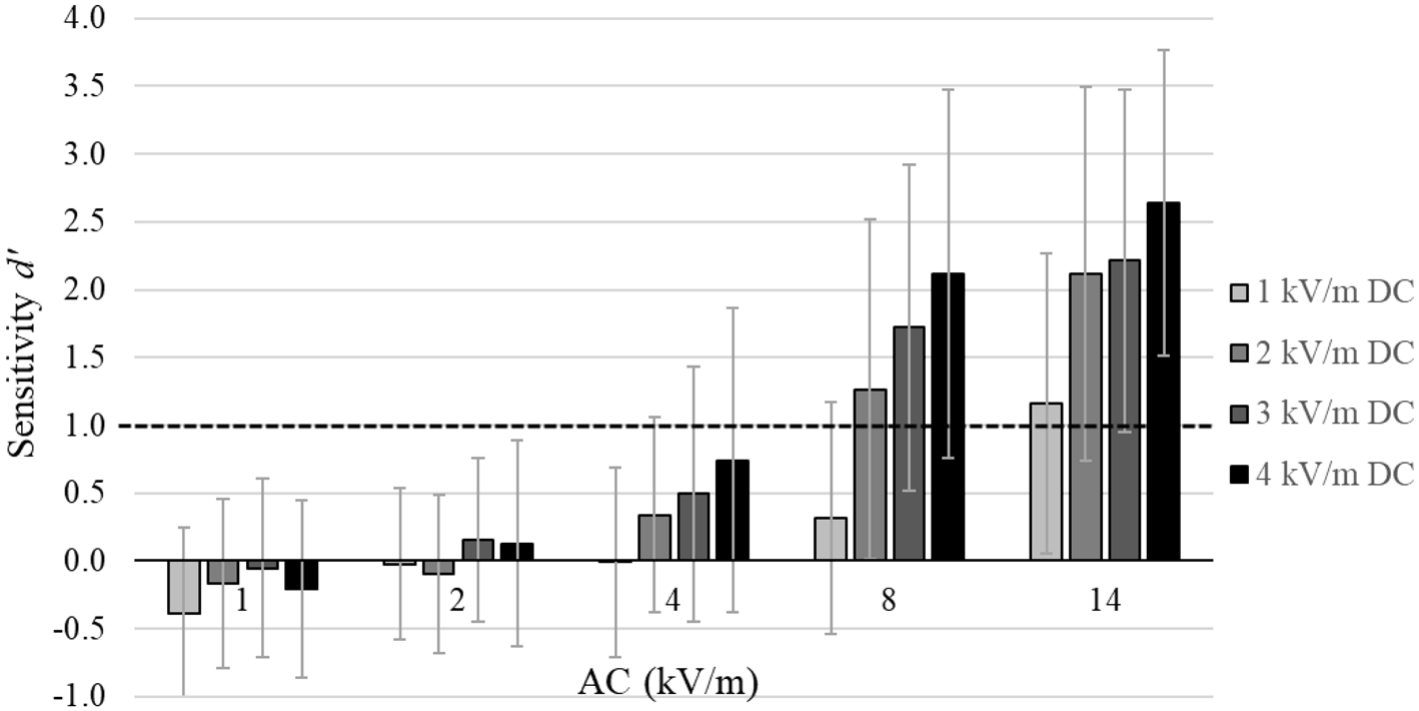
Influence of the DC component on AC sensitivities. Averaged sensitivities (d′) for DC EF strengths of 1, 2, 3, and 4 kV/m combined with AC EF strengths of 1, 2, 4, 8, and 14 kV/m for n = 49 participants. Bars reflect standard deviations. Average performances exceeding the black dotted line are associated with successful detection (i.e., *d*′ ≥ 1).

Rm ANOVA results are displayed in Table 2. As a result of the significant interaction effect, rm ANOVAs for every AC EF strength revealed significant effects for 1, 4, 8, and 14 kV/m [(F(3, 46) = 3.05, *p* < 0.05, η_p_^2^ = 0.17), (F(3, 46) = 5.59, *p* < 0.01, η_p_^2^ = 0.27), (F(3, 46) = 25.14, *p* < 0.001, η_p_^2^ = 0.62), (F(3, 46) = 22.39, *p* < 0.001, η_p_^2^ = 0.59)], but not for 2 kV/m (*p* = 0.13). For every DC EF strength, a significant AC effect was found for levels 1, 2, 3, and 4 kV/m [(F(4, 45) = 17.32, *p* < 0.001, η_p_^2^ = 0.61), (F(4, 45) = 31.72, *p* < 0.001, η_p_^2^ = 0.74), (F(4, 45) = 48.3, *p* < 0.001, η_p_^2^ = 0.81), (F(4, 45) = 65.41, *p* < 0.001, η_p_^2^ = 0.85)]. As depicted in Fig. 1, the DC component significantly influences detection performance, which is most salient when AC EF strength was 8 or 14 kV/m. Additionally, AC EF strengths below 8 kV/m were not sufficient to facilitate EF detection independent of the DC component. Both, DC and AC EF increases, influenced the number of participants with successful EF detection (Table 3). Interestingly, one participant was able to detect even the lowest EF combination of 1 kV/m DC and 1 kV/m AC.

**Table 2:** Results of the 4 × 5 rm ANOVA with factors *DC EF strength* (1, 2, 3, and 4 kV/m) and *AC EF strength* (1, 2, 4, 8, and 14 kV/m).

Factor	df	F	*p*	η_p_^2^
DC EF strength	3144	37.21	< 0.001	0.44
AC EF strength	4192	156.53	< 0.001	0.77
DC EF strength*AC EF strength	12,576	10.75	< 0.001	0.18

**Table 3:** Overall numerical distribution of participants (out of n = 49) successfully detecting a specific hybrid EF (*d*′ ≥ 1).

DC (kV/m)	AC (kV/m)				
	1	2	4	8	14
1	1	1	2	8	25
2	1	2	12	25	40
3	2	5	9	34	40
4	1	6	15	38	44

**Figure 2:**
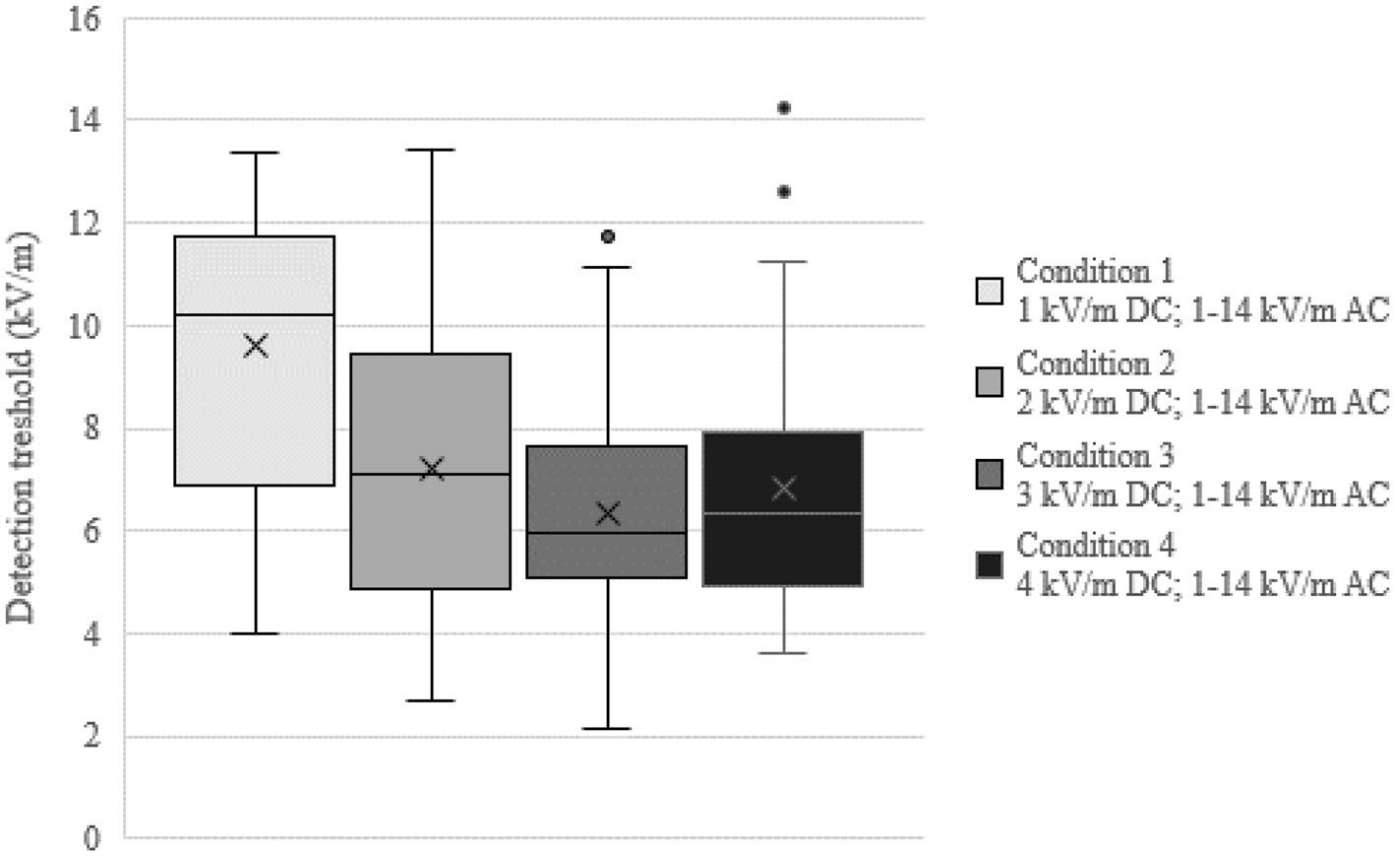
Average detection thresholds for all test conditions. AC EF strengths per condition were 1, 2, 4, 8, or 14 kV/m; DC component: 1 kV/m in Condition 1, 2 kV/m in Condition 2, 3 kV/m in Condition 3, and 4 kV/m in Condition 4. Number of participants were 24, 33, 37, and 44 for Condition 1, 2, 3, and 4, respectively. Crosses mark averaged detection thresholds. Median values are indicated by the horizontal bar within the boxes. Minima and maxima are indicated by whiskers except outliers marked by dots. Number of participants where detection thresholds could be estimated for all conditions was n = 14.

Detection thresholds computed on total EF strengths were 9.6 kV/m (SD = 2.61), 7.19 kV/m (SD = 2.76), 6.35 kV/m (SD = 2.14), and 6.83 kV/m (SD = 2.38) for test conditions 1, 2, 3, and 4, respectively. Values were entered into an rm ANOVA showing a significant effect (F(3, 11) = 9.84, *p* < 0.01, η_p_^2^ = 0.73). Pairwise comparisons of the test conditions showed significant differences between all adjacent levels (all p’s ≤ 0.05). As visible in Fig. 2, detection thresholds decreased from test condition 1 to 2 as well as from test condition 2 to 3, whereas in test condition 4 a slight increase of average detection threshold was observed.

**Figure 3:**
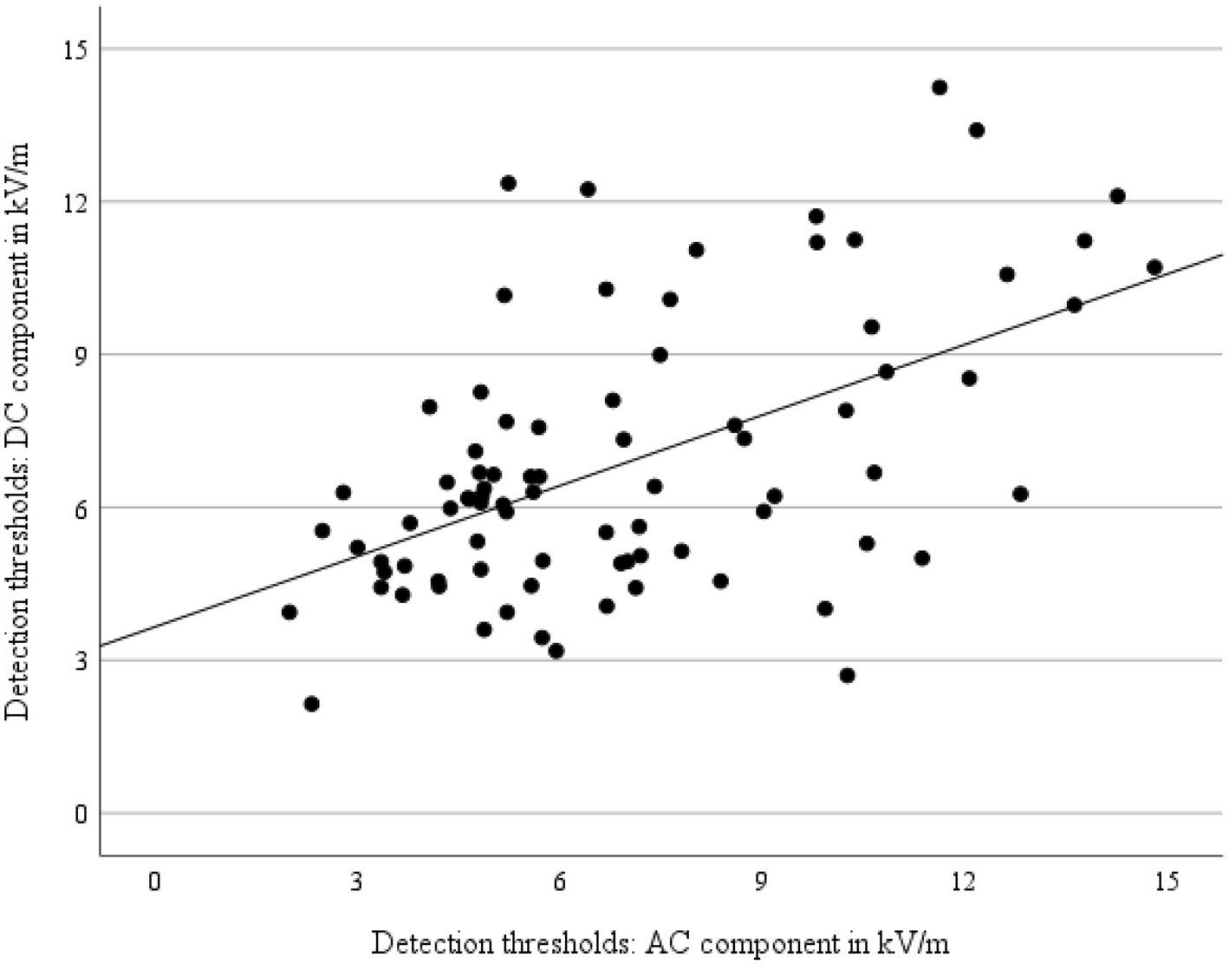
Linking of the detection thresholds of total EFs from the current study (DC component) and a former study (AC component; Jankowiak et al.^7^). Due to methodical constraints, i.e., participants showing non-plausible trajectories in *d*′-values, the number of cases was limited to 15, 20, 23, and 25 for test conditions 1, 2, 3, and 4 leading to a total case number of 83.

Detection thresholds of participants in the current study and the former study^7^ were compared using correlational analyses. A significant correlation between the detection thresholds of total EFs of both studies of *r* = 0.54, *p* < 0.001 was found. On average, good performance in EF detection in the current study was associated with good performance in our former study^7^, as illustrated in Fig. 3.

## Discussion

The study aimed to investigate how small changes of the DC component influence the human perception of AC–DC hybrid EFs. Therefore, 49 participants with very good EF detection ability were exposed with hybrid EFs, whereby the DC component was varied between 1 and 4 kV/m within four test conditions and co-exposed with AC EFs between 1 and 14 kV/m. It was shown that total EF detection thresholds decreased with increasing DC component, whereas even the lowest hybrid EF combination was detected by one participant. The study expands on the findings of Jankowiak et al.^7^, showing that the AC component has a major influence on hybrid EF detection.

Average sensitivity values increased with increasing DC and AC EF strength, which is only visible in combinations with AC EF strengths of 8 and 14 kV/m. Since differences in sensitivities of *d*′ < 1 cannot be interpreted, it can be inferred that no EF detection occurred on group average at AC EF strength of 1, 2, and 4 kV/m, regardless of the DC component. In contrast, on a numerical level, one participant was able to detect even the lowest EF combination of 1 kV/m DC and 1 kV/m AC. Interestingly, increasing the DC component does not increase the number of participants successfully detecting hybrid EFs when AC EF strength was 1 kV/m. Comparing to Jankowiak et al.^7^, the distribution of the sensitivity values across test conditions was similar, whereas in the former study, an AC component of 1–4 kV/m was combined with varying DC EF strengths. The overall numerical distribution of participants successfully detecting a specific hybrid EF was also similar ranging from 1 to 44 in the current study (n = 49) and from 1 to 43 in the former study (n = 51, Jankowiak et al.^7^). Nevertheless, on a detail level, eleven participants of the former study could successfully detect 1 kV/m DC and 4 kV/m AC, whereas only two participants were able to detect this EF combination in the current study. This difference might be explained by a different context defined by the dominant EF. Whereas in the former study, participants were required to detect DC EFs (combined with low AC EFs), in the current study the major EF component was AC (combined with low DC EFs).

Detection thresholds of total EFs overall decreased with an increasing DC component. Yet, in test condition 4, a slight change in the direction of this trend was observed. This effect can be mainly attributed to outliers ranging between 12 and 15 kV/m (see Fig. 2). These outliers in test condition 3 and 4 might reflect a part of a group at the upper end of the distribution performing not as good in AC-dominant EF as in DC-dominant EF, which was applied in the former study^7^. Compared to Jankowiak et al.^7^, detection thresholds were similar with 9.6 kV/m (former: 8.89 kV/m), 7.19 kV/m (former: 7.82 kV/m), 6.35 kV/m (former: 6.48 kV/m), and 6.83 kV/m (former: 5.7 kV/m) for test conditions 1, 2, 3, and 4, respectively. A DC component of 1, 2, and 3 kV/m is associated with a significant decrease of detection thresholds, whereas when DC EF reaches 4 kV/m no further decrease of average detection threshold was observed, which might be explained by individual performance differences in AC- and DC-dominant EFs.

Correlational analyses of participants’ performance in the current and the former study^7^ revealed a significant coherence. In general, this reliability strengthens the results of both investigations. Along with the finding that participants follow a linear relation of detection performance between both studies, deviations in two directions were observed: (1) participants with good performance in the current study and low performance in the former study, and (2) participants with low performance in the current and good performance in the former study. The main difference between both studies was the proportion of DC and AC in the presented hybrid EFs. Whereas DC EF was dominant in the former study, AC EF was the major EF in the current one. As explained earlier, DC EF perception is likely associated with conductive processes, whereas AC EF perception is driven by mechanical force on body hairs^3,4^. This distinction can explain different performance levels of participants since body hairs are very different on an individual level. It is therefore reasonable to derive that due to individual body hair characteristics, performance level varied depending on the proportion of DC and AC EF.

### Limitations

The transferability of the current results to population cannot be done since only participants with an above-average ability to detect hybrid EFs were included to obtain a most homogenous cohort. Furthermore, due to safety reasons, participants were grounded during experiments. Therefore, perception under real conditions might be different from the laboratory setup applied in the current study. When computing detection thresholds, participants showing implausible trajectories at least in one test condition were excluded from the overall analysis, leading to n = 14 participants included in the rm ANOVA on detection thresholds. Analyses of *d*′ as well as detection threshold data depicted in Fig. 2 are not affected by this limitation.

### Implications

The significant correlation found between the detection performances in the current study and a previous study^7^ underscores the robustness of results in EF detection research. However, differences in performance between the two studies were observed and can be attributed to variations in the proportions of DC and AC components. While the detection thresholds of most participants were similar in both studies, the distribution of some participants' performance differed, potentially due to individual factors such as body hair. Further research focusing on individual factors is needed to better understand these mechanisms.

The results of the current study clearly demonstrate the influence of the DC component on human hybrid EF perception, as evidenced by a decrease of total EF detection thresholds with increasing DC EF strength. In the construction of overhead power lines, this outcome might help designing the pylon geometry in terms to prevent sensory processes in humans living or working in close proximity. When combined with the outcome of Jankowiak et al.^7^, it can be concluded that both the ascending DC and AC components significantly lower total EF detection thresholds. Thus, both components should be considered equally in the construction process. The current results can help realizing the energy transition by providing guideline values of EF perception of a highly sensitive cohort.

## Data Availability

The datasets used and/or analyzed during the current study is available from the corresponding author on reasonable request.
